# Identification of hub mRNAs and long non-coding RNAs involved in temozolomide-resistant glioblastoma (brain cancer) cell lines

**DOI:** 10.1007/s12672-026-05243-2

**Published:** 2026-06-05

**Authors:** Mostafa Alamholo, Alireza Tarinejad

**Affiliations:** 1 Department of Biotechnology and Biomedicine, Institute of Science and Modern Technology, Rojava University, Qamishlo, Syria; 2 Department of Biochemistry, Faculty of Natural Science and Technology, Rojava University, Qamishlo, Syria; 3 Department of Biotechnology, Agriculture Faculty, Azarbaijan Shahid Madani University, Tabriz, Iran

**Keywords:** Glioblastoma, Hub gene, LncRNAs, Therapeutic targets

## Abstract

**Purpose:**

Temozolomide (TMZ) is used to treat glioblastoma cancer and it is essential to identify key genes and investigate the molecular mechanisms of glioblastoma cancer cells resistant to temozolomide.

**Materials and methods:**

Raw RNA-Seq data from glioblastoma cell lines were analyzed by Linux and used to align to the human reference genome GRCh38 using HISAT2 as well as edgeR in the R package was used. KEGG and EnrichR pathways were used for enrichment analysis and degree level in Cytoscape was used to identify hub genes.

**Results:**

The top up-regulated genes included NeuroD4, IgSF21, PCDH8, ACKR1, and TC2N, and top down-regulated genes including ITGAX, SIGLEC-15, CCL8, MOG, PENK, and SELE were identified. The genes up-expressed in the biological process (BP) were mostly related to regulation of phospholipase c-activating G protein-coupled receptor signaling pathway, in the molecular function (MF) belonged to calcium- dependent cysteine-type endopeptidase activity, and in the cellular component (CC) were associated with actin filament. Next, the genes down-expressed in the BP were mainly related to cytokine-mediated signaling pathway, in the MF related to chemokine receptor binding, and in the CC were belonged to MHC Class II protein complex. Key genes related to KEGG pathways mainly were shown chemokine, inflammations, apoptosis, and cell adhesion functions. Top up-regulated TFs including TFAP2C, and BCL11A and top down-regulated TFs such as IRF3, and CEBPB were identified. Top up-regulated hub genes including NEUROD1, DCX, and ACKR1 and top down-regulated hub genes such as IL1B, CXCL10, and ITGAX were identified. Top up-regulated lncRNAs including NAV2-AS2, CAMTA1-AS2, and MEIS1-AS3, and top down-regulated lncRNAs such as MIR3142HG, SUGCT-AS1, and DBH-AS1 were identified. Finally, this study was evaluated with qRT-PCR results of reported studies and the expression of down and up-regulated genes was confirmed.

**Conclusion:**

Accordingly, these genes can be incorporated into clinical prognostic models and provide panels of genes for selecting personalized and appropriate therapeutic approaches. Overall, key genes can be suggested as influential genes associated with temozolomide-resistant glioblastoma cell lines.

**Supplementary Information:**

The online version contains supplementary material available at 10.1007/s12672-026-05243-2.

## Introduction

Glioblastoma (brain cancer) is the most common and aggressive primary tumor in the adult brain. The median survival rate for patients with glioblastoma is still 16–18 months [1]. Glioblastomas are cytogenetically heterogeneous tumors and are found mostly in the cerebral hemispheres, especially in the frontal and temporal lobes, while only a small percentage of them occur in the cerebellum, brainstem and spinal cord [2]. The main reasons why glioblastoma is challenging to treat are related to the limited surgical removal of the tumor its resistance to radiation and chemotherapy [3]. Due to their aggressive migrative behavior, glioblastoma cells have the ability to invade normal brain tissue, and infiltrate the brain parenchyma, and glioblastoma cells escape complete surgical removal and, therefore, the tumor usually re-appears within a few centimeters of its original location [4].

Traditional medications such as temozolomide (TMZ), an oral alkylating agent used to treat glioblastoma and is a first-line chemotherapy drug for glioblastoma. TMZ exerts an antitumor effect via methylation of DNA at the O^6^ position of guanine, forming O^6^-methylguanine that incorrectly pairs with thymine. This base mispairing induces continuous activation of the mismatch repair (MMR) pathway and DNA double strand breaks (DSBs), which eventually results in cell cycle arrest and apoptosis [5]. Radio chemotherapy with TMZ, followed by six courses of adjuvant TMZ and preceded by surgery with maximal safety, is still the first line treatment for glioblastoma today. Despite multimodality treatment that includes surgery, radiotherapy, and chemotherapy using the DNA alkylating agent temozolomide, the median patient survival is only 15 months [6]. Unfortunately, the survival benefit provided by TMZ is very small and many patients do not respond to this treatment and intrinsic or acquired resistance to TMZ has been extensively studied [7].

Drug resistance is a major factor in glioma recrudescence and death. TMZ-resistant U251 cells exhibited increased microRNA, and STAT3/p-STAT3 expression, and decreased mitochondrial DNA copy number [8]. Immunoglobulin superfamily member 21 (IgSF21) selectively regulates inhibitory presynaptic differentiation through interacting with presynaptic neurexin2α and plays a crucial role in synaptic inhibition in the brain [9]. Protocadherin 8 (PCDH8) functions as a calcium-dependent adhesion protein play an important role in its prognosis, immune infiltration, and diagnosis PCDH8 suppressed glioma cell proliferation, and that the loss of PCDH8 may stimulate the proto‑oncogene Wnt/β‑catenin signaling pathway and therefore promote glioma cell proliferation [10]. E-selectin (SELE) is expressed in endothelial cells and plays an important role in inflammation by facilitating the adhesion and migration of immune cells to sites of inflammation and its expression levels are associated with more aggressive tumors and poorer prognosis in cancer patients [11]. Polymeric immunoglobulin receptor (PIGR) is a crucial receptor that primarily mediates the transcytosis of immunoglobulins A and M across epithelial cells and is an emerging therapeutic target with significant potential for the development of novel cancer treatment strategies [12]. Proenkephalin (PENK) is distributed extensively throughout the central nervous system (CNS), including the cerebral cortex, and basal ganglia. The therapeutic potential of proenkephalin-derived peptides is underscored by gene therapy approaches for chronic pain and their involvement in neurological and psychiatric disorders [13]. Atypical chemokine receptor 1 (ACKR1) is a key regulator that binds chemokines involved in inflammatory responses and cancer proliferation, angiogenesis, and metastasis [14].

Transcription factors (TFs) are proteins with DNA binding domains that bind to the specific sequences of DNA in the promoter and/or enhancer region to regulate target gene transcription. Cancer is a multi-step process and requires constitutive expression/activation of TFs for growth and survival. Many of the TFs reported so far are critical for carcinogenesis. PDIA3P1 is responsible for resistance to TMZ in multiple glioblastoma cell lines and is associates with CEBPB as a TF and stabilizes it by preventing its ubiquitination by the MDM2 E3 ubiquitin ligase [15]. The epidermal growth factor (EGF-2) as a domain is the most frequently altered gene in glioblastoma, which plays an important role in tumor development and anti-tumor immune response [16].

Long non-coding RNAs (lncRNAs) have been reported to involve in diverse biological processes, including tumor immunity. LncRNAs include a class of transcripts that are more than 200 nucleotides long, and have many regulatory mechanisms in addition to their non-protein-coding potential. A growing number of reports have shown that lncRNAs play a critical role in cancer progression such as glioblastoma. Abnormal expression of ncRNA is associated with tissue fibrosis, cancer and cardiovascular diseases [17]. Some lncRNAs have been implicated in glioma proliferation, apoptosis, motility and stemness maintenance. CSE1L-DT (chromosome segregation 1-Like divergent transcript) protein is located on 20q13.13, and is associated with microtubules and mitotic spindles, and is involved in the progression of tumor, proliferation of cell, and apoptosis. Studies show that CSE1L is a potential oncogene that is highly expressed in colorectal, ovarian and gastric tumors [18]. Furthermore, limb expression antisense RNA 1 (LIX1-AS1) lncRNA is involved in cancer development and progression and could be used as a target for design of drug in cancer treatment [19]. MEIS proteins are historically associated with tumorigenesis, metastasis, and invasion in cancer. Gene expression analysis of glioma and glioblastoma cells revealed that Meis2 was one of the differentially expressed genes and it could be a prognostic biomarker for glioma and glioblastoma development. MEIS2 has been shown to be essential for neuroblastoma cell survival and proliferation and also may act as oncogenes in neuroblastoma [20].

The sensitivity of drugs against malignant tumors and the development of resistant cancer cells causes high mortality rate in the world, and a major part of this problem is related to the expression and silencing of key genes. Genomic and chip data are becoming increasingly abundant owing to advance in sequencing methods. Therefore, it is essential to understand the molecular mechanisms and genes involved in the resistance of cancer cells and tumors to drugs. The aim of this study was to identify differentially expressed genes (DEGs), LncRNA, hub genes, and molecular mechanism through bioinformatics analysis of raw RNA-seq data from normal and temozolomide -treated samples of three different glioblastoma cell lines.

## Materials and methods

### RNA sequencing (RNA-Seq) data analysis

Raw RNA-Seq data from glioblastoma cells (GSE225869, GSE249544, and GSE253464) were imported directly from the SRA database via the Gene Expression Omnibus database (GEO, http://www.ncbi.nlm.nih.gov/geo) (Table 1). First, the SRA files were downloaded from the SRA database using “./prefetch” tool, then converted to FASTQ files using “./fastq-dump” tool from SRAtoolkit. RNA-Seq read quality via FastQC and trimming of FASTQ files were performed with 123FASTQ (Table 1). After quality checking and removal of technical sequence contaminations, FASTQ files trimmed which had been corrected by alignment with the human reference genome (GRCh38) using HISAT2 were converted to SAM files. HISAT2 is a bioinformatics tool designed to facilitate rapid and sensitive alignment of transcriptome reads to the reference genome. Next, the SAM format files converted to BAM format using the “samtools view” tool and then, BAM files converted to sorted BAM files using " samtools sort " tool. Finally, to quantify gene expression in each sample (eight temozolomide -treated glioblastoma cancer cells and eight control samples), the featureCounts tool was used using “./featureCounts” via the gtf file mapping [21]. The results were normalized via the TMM method and cluster dendrogram was drawn for samples (supplementary file Fig. 1).

**Fig. 1:**
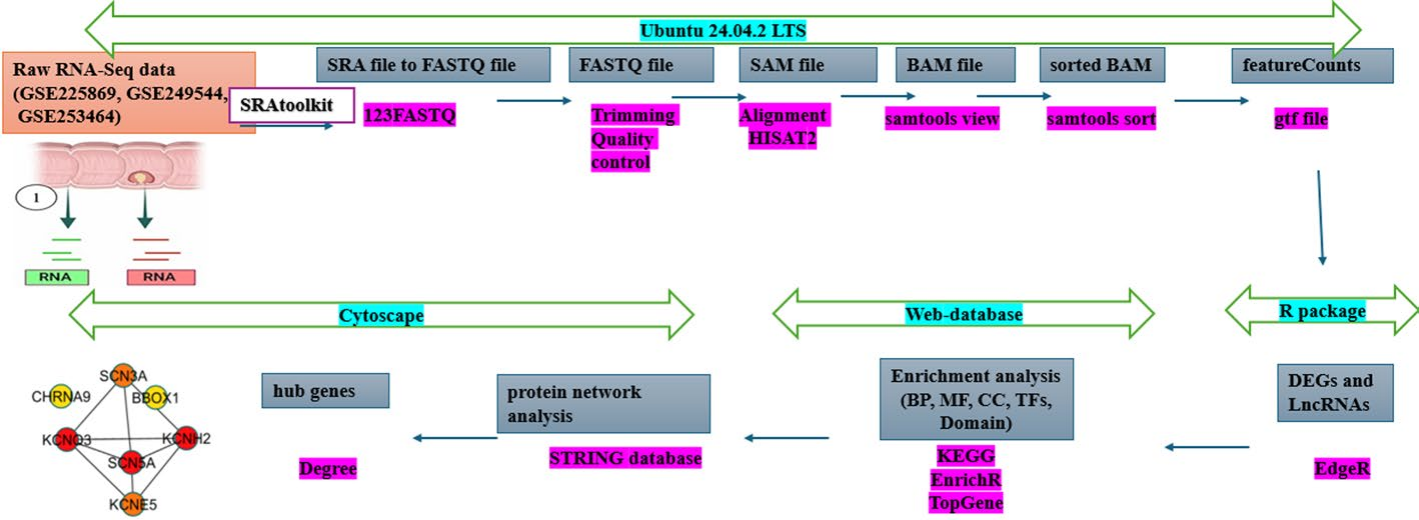
The workflow shows a step-by-step and comprehensive review of the analysis of this study

DEGs were performed and identified using edgeR in the R package. Log2foldchange (Log2FC ≥ 2 and Log2FC ≤ − 2) and p-values ≤ 0.01 were used to filter the up and down-regulated genes as well as to identify LncRNA. Finally, a heatmap of the most significant genes, |log2FoldChange|> 4, and p-value < 0.01 was created using pheatmap. The workflow of our study approaches is show (Fig. 1).

**Table 1:** RNA seq samples from multiple glioblastoma cell lines

GSE	GSM	SRA run ID	Type of samples	Cell line
GSE225869	GSM7058988	SRR23592479	Control	U251
	GSM7058989	SRR23592478	Control	
	GSM7058990	SRR23592477	Control	
	GSM7058994	SRR23592473	TMZ treated	
	GSM7058995	SRR23592472	TMZ treated	
	GSM7058996	SRR23592471	TMZ treated	
GSE249544	GSM7949496	SRR27118226	Control	CSCHF2303
	GSM7949508	SRR27118214	Control	
	GSM7949499	SRR27118223	TMZ treated	
	GSM7949511	SRR27118211	TMZ treated	
GSE253464	GSM8021070	SRR27592763	Control	BT935
	GSM8021071	SRR27592762	Control	
	GSM8021072	SRR27592761	Control	
	GSM8021073	SRR27592760	TMZ treated	
	GSM8021074	SRR27592759	TMZ treated	
	GSM8021075	SRR27592758	TMZ treated	

### Enrichment analysis

To identify important pathways, KEGG (Kyoto Encyclopedia of Genes and Genomes) databases was used for up and down-regulated genes (https://www.genome.jp/kegg/mapper/color.html). KEGG is a database resource for representation and analysis of biological systems. Pathway maps are the primary dataset in KEGG representing systemic functions of the cell and the organism in terms of molecular interaction and reaction networks [22]. Moreover, the EnrichR (https://maayanlab.cloud/Enrichr/) database was used to analyze biological processes (BP), molecular functions (MF), and cellular components (CC), as well as identify of domains. Pathways were considered significant if the p-value was ≤ 0.01 [23]. The TopGene (https://toppgene.cchmc.org/enrichment.jsp) database was used to identify TFs.

### Hub gene network analysis

Protein–protein interactions (PPIs) were performed through the String protein database plugin (https://string-db.org/) in Cytoscape version 3.6.1 to identify protein networks associated with the identified genes. The STRING database provides information on predicted and known PPIs and Cytoscape is a powerful tool for visualizing complex networks of molecular interactions. The hub genes in the biological network were identified using degree level in Cytoscape [24].

### Confirmation of key genes using reported qRT-PCR studies

To confirm the results of the present study, key genes with high expression for each of the up and downregulated genes related to TMZ glioblastoma cell lines were compared with reported transcriptomics data including qRT-PCR studies.

## Results

### Up and down‑regulation of candidate genes

**Fig. 2:**
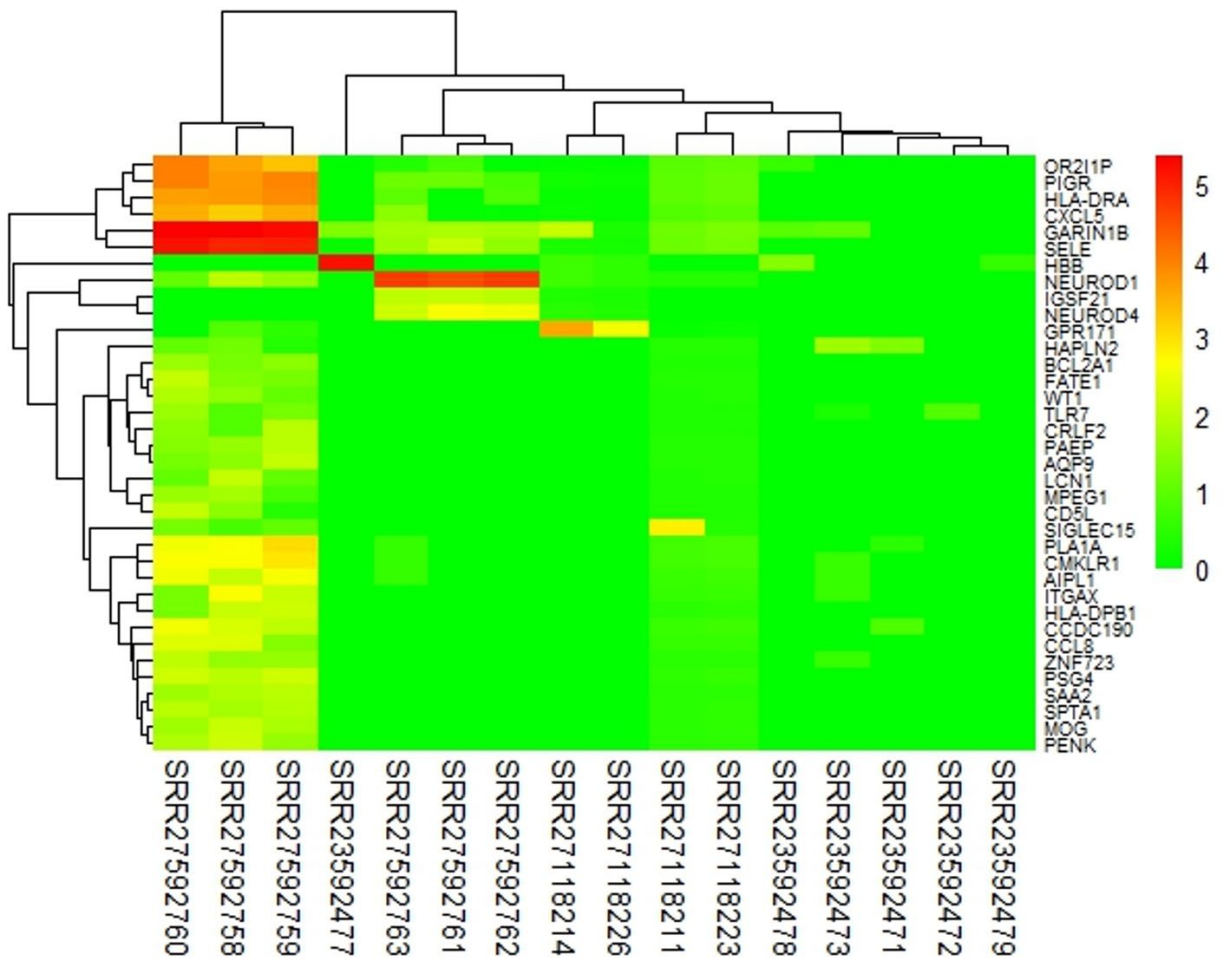
Presentation of important genes with high expression from normal and temozolomide -treated samples of three different glioblastoma cell lines using a heatmap

Top up and down-regulated genes (Log2FC ≥ 2 and Log2FC ≤ − 2) and p-values ≤ 0.01 involved in temozolomide-resistant glioblastoma different cell lines are shown in Table 2. Accordingly, 118 up-metaDEGs and 278 down- metaDEGs were identified. The up-regulated genes with high expression level including NeuroD4, IgSF21, PCDH8, TRIM31, ACKR1, Mep1b, and TC2N and down-regulated genes such as ITGAX, SIGLEC-15, CCL8, MOG, PENK, SELE, and PIGR were identified. To better visualize the DEGs patterns, a heatmap of the top up-regulated and down-regulated genes was created (Fig. 2).

**Table 2:** Top 15 key genes with differential expression

Ensemble gene ID	Gene_Symbol	Description	logFC	PValue
Up-regulated genes				
ENSG00000123307	NEUROD4	Neuronal differentiation 4	5.732761672	0.0003079214
ENSG00000117154	IGSF21	Immunoglobin superfamily member 21	5.006080247	0.0002921285
ENSG00000136099	PCDH8	Protocadherin 8	3.821166405	0.0008572215
ENSG00000125255	SLC10A2	Solute carrier family 10-member 26	3.668685422	0.0006395235
ENSG00000213088	ACKR1	Atypical chemokine receptor 1	3.636209368	0.0001080826
ENSG00000204616	TRIM31	Tripartite motif containing 31	3.367775665	0.0027567663
ENSG00000104059	ENTREP2	Endosomal transmembrane epsin interactor 2	3.104512332	0.0024652161
ENSG00000141434	MEP1B	Meprin A subunit beta	3.049524609	0.0027130751
ENSG00000165929	TC2N	Tandem C2 domains, nuclear	3.026119885	0.002865877
ENSG00000089169	RPH3A	Rabphilin 3 A	2.929884577	0.002478145
ENSG00000172137	CALB2	Calbindin 2	2.907378154	0.008953829
ENSG00000260903	XKR7	XK related 7	2.896085268	0.005291312
ENSG00000179915	NRXN1	Neurexin 1	2.815621423	0.001313042
ENSG00000186439	TRDN	Triadin	2.794608279	0.002624835
ENSG00000105675	ATP4A	ATPase H+/K+ transporting subunit alpha	2.66914098	0.002495115
Down-regulated genes				
ENSG00000140678	ITGAX	Integrin subunit alpha X	-5.387255305	0.000805253
ENSG00000197046	SIGLEC15	Sialic acid binding Ig like lectin 15	-5.255932163	0.003032038
ENSG00000108700	CCL8	C-C motif chemokine ligand 8	-5.251698734	0.001723919
ENSG00000243137	PSG4	Pregnancy specific beta-1-glycoprotein 4	-5.246084172	0.001499289
ENSG00000204655	MOG	Myelin oligodendrocyte glycoprotein	-4.9371292	0.003072221
ENSG00000174600	CMKLR1	Chemerin chemokine-like receptor 1	-4.936083246	0.000378262
ENSG00000181195	PENK	Proenkephalin	-4.909711497	0.003098271
ENSG00000134339	SAA2	Serum amyloid A2	-4.832179857	0.003459448
ENSG00000268696	ZNF723	Zinc finger protein 723	-4.822595667	0.002389825
ENSG00000007908	SELE	Selectin E	-4.785590148	0.000920956
ENSG00000132702	HAPLN2	Hyaluronan and proteoglycan link protein 2	-4.562517885	0.001553043
ENSG00000147378	FATE1	Fetal and adult testis expressed 1	-4.497996074	0.008213618
ENSG00000129221	AIPL1	Aryl hydrocarbon receptor interacting protein like 1	-4.468129991	0.001119907
ENSG00000162896	PIGR	Polymeric immunoglobulin receptor	-4.438573911	0.000469349
ENSG00000205755	CRLF2	Cytokine receptor like factor 2	-4.328594928	0.010634715

### Gene ontology (GO) and pathway analysis

**Fig. 3:**
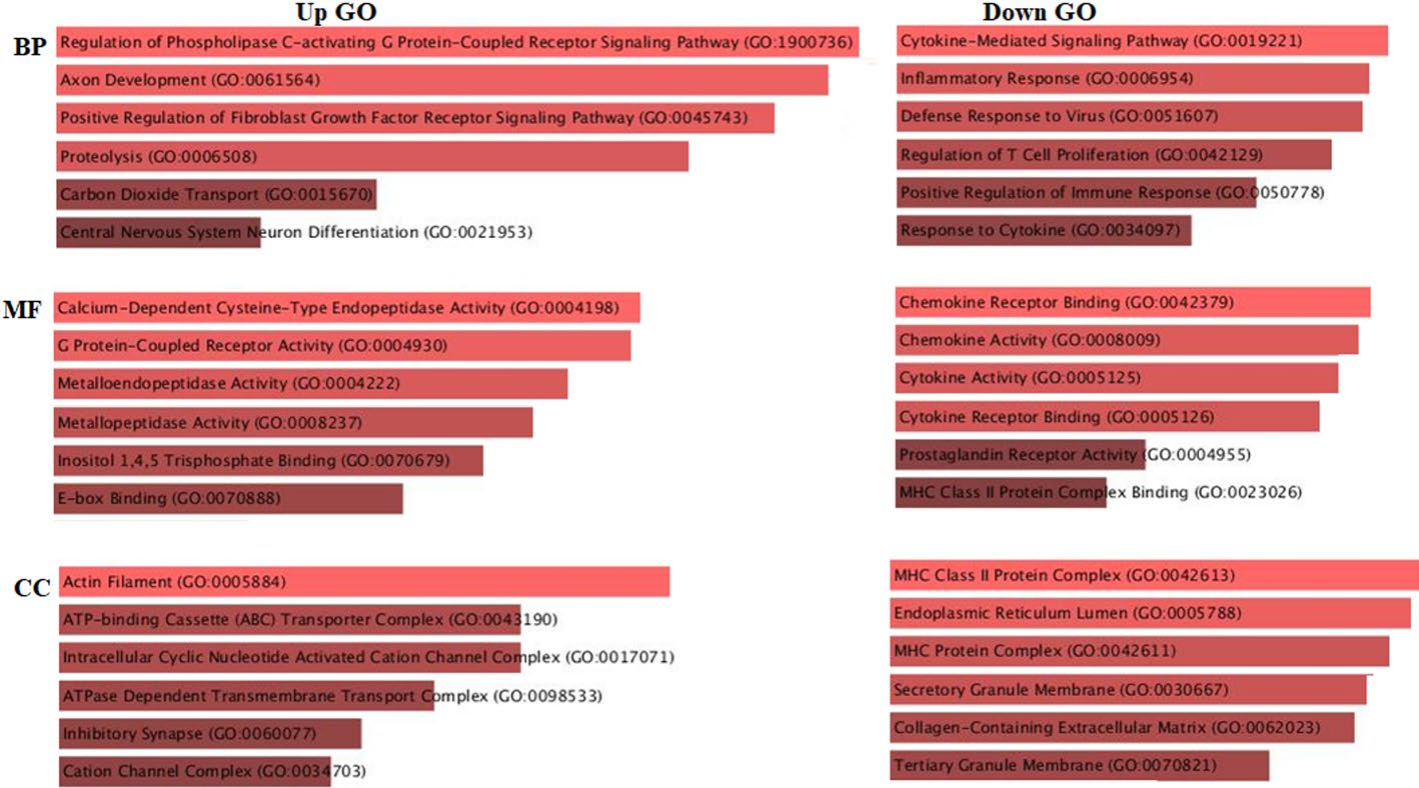
The most significant changes in BP, MF and CC in relation to up and down-regulated genes based on GO enrichment analysis

The top terms of BP, MF, and CC in relation to up and down-regulated genes are shown in Fig. 3. Based on the results of the EnrichR database, the up-regulated genes in BP were mainly related to regulation of phospholipase C-activating G protein-coupled receptor signaling pathway (GO:1900736), axon development (GO:0061564), and positive regulation of fibroblast growth factor receptor signaling pathway (GO:0045743), in MF were belonged to calcium- dependent cysteine-type endopeptidase activity (GO:0004198), and G protein-coupled receptor activity (GO:0004930) and in CC were rich in the actin filament (GO:0005884), and ATP-binding cassette (ABC) transporter complex (GO:0043190). Next, the down-regulated genes in BP were associated with cytokine-mediated signaling pathway (GO:0019221), inflammatory response (GO:0006954), and positive regulation of immune response (GO:0050778), in MF belonged to chemokine receptor binding (GO:0042379), and chemokine activity (GO:0008009), and in CC enriched in MHC Class II protein complex (GO:0042613).

Next, key biological pathways through KEGG database including cytokine-cytokine receptor interaction, IL-17 signaling pathway, JAK-STAT signaling pathway, and chemical carcinogenesis were identified. Accordingly, key genes related to these pathways mainly were shown chemokine, inflammations, and apoptosis, cell cycle inhibition and cell adhesion functions (Table 3).

**Table 3:** Top key KEGG pathways related to up and down-regulated genes

Pathways	Key genes	Function
Cytokine-cytokine receptor interaction	CCL5, IL6, and IL7 as down genes	Chemokine
IL-17 signaling pathway	IL6, and IL1B, CXCL20, CXCL8 as down genes	Inflammations
JAK-STAT signaling pathway	IL2, CXCL10, IL4, CNTF, CTF1, CLCF, CSF1, and CSH2 as down genes	Cell-cycle inhibition
Apoptosis	CASP1, HRK, and TRAF1 as down genes	Pro-apoptosis and pro-survival
Chemical carcinogenesis	DLL3 as up gene, CYP1A1 as down gene	Chemical carcinogenesis
Hormone signaling	ADCY1 as down gene	Brain development
Proteoglycans in cancer	VAV3 as up gene, COL1A1 and CDKN1A as down genes	Cell adhesion

### TFs and domains of up and down-regulated DEGs

Top TFs and domains of up and down-regulated DEGs are shown in Table 4. Accordingly, top TFs including TFAP2C, BCL11A, NR4A1 and NOTCH1 related to up-regulated genes and IRF3, SMAD4, CEBPB, and NFE2L2 related to down-regulated genes were identified. Moreover, key domains such as leu-rich_rpt, zinc_protease, BHLH and cadherin related to up-regulated genes as well as Ig-like_fold, EGF_2, chemokine_IL8, thyroglobulin_1, and MHC_II_a/b_N associated with down-regulated genes were detected.

**Table 4:** Top TFs and domains of up and down-regulated DEG with p-value < 0.01

Up-regulated genes					Down-regulated genes		
TF		Domain		TF		Domain	
	ID	Name	p-value		ID	Name	p-value
TFAP2C	IPR001611	Leu-rich_rpt	4.49E-03	IRF3	IPR013783	Ig-like_fold	4.18E-04
BCL11A	PS00142	Zinc_protease	2.39E-04	SMAD4	PS01186	EGF_2	6.97E-09
NOTCH1	PF00010	HLH	4.26E-04	CEBPB	IPR007110	Ig-like_dom	7.84E-04
NR4A1	PS50888	BHLH	5.42E-04	NFE2L2	IPR001811	Chemokine_IL8	8.46E-09
LMO4	PF08266	Cadherin	2.35E-03	NR4A1	PF00048	IL8	8.46E-09
PDX1	IPR000372	LRRNT	5.09E-04	NFKB1	PS50234	VWFA	1.96E-05
LMO2	IPR022683	Calpain	3.77E-05	SRF	PF00086	Thyroglobulin_1	7.80E-05
ATF3	IPR022575	Neurogenic_DUF	1.90E-04	CBFB	PS51470	FG_GAP	2.27E-04
TCF4	PF03607	DCX	6.58E-04	CTNNB1	IPR014745	MHC_II_a/b_N	8.86E-04
SUZ12	PF08686	PLAC	2.78E-03	MYC	SM00126	IL6	1.96E-04

### PPIs and gene network analysis to identify candidate hub genes

**Fig. 4:**
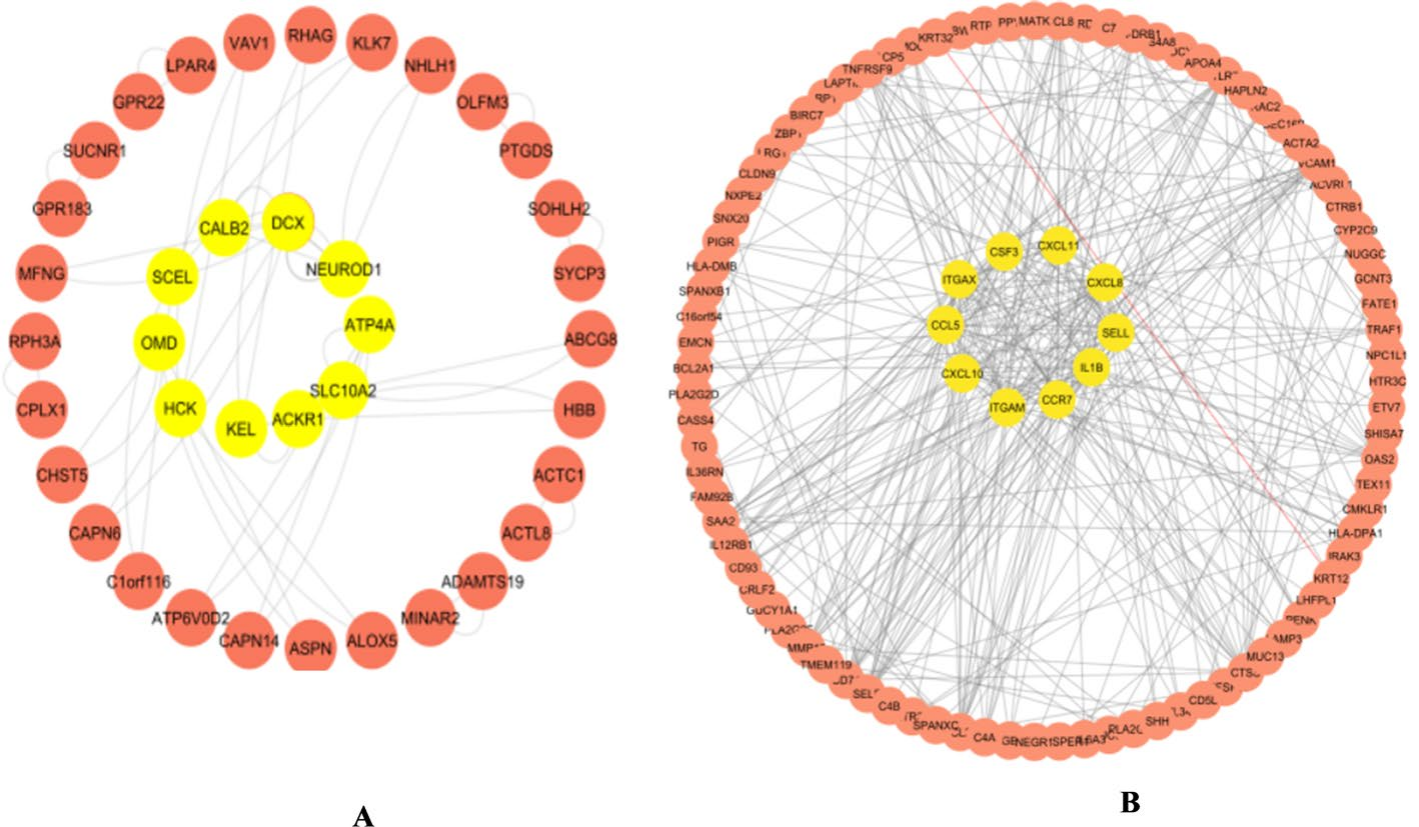
Identification of top up and down-regulated candidate hub genes (yellow color) through PPI network analysis. **A**: up-regulated hub genes; **B**: down-regulated hub genes

Top down and up-regulated hub genes are shown in Table 5; Fig. 4. Accordingly, up-regulated hub genes with high degree mainly related to NEUROD1 (neuronal differentiation 1), DCX (doublecortin), ATP4A (ATPase H+/K+ transporting subunit alpha), ACKR1 (atypical chemokine receptor 1), and HCK (HCK proto-oncogene) as well as down-regulated hub genes included IL1B (interleukin 1 beta), CXCL8 (C-X-C motif chemokine ligand 8), CXCL10 (C-X-C motif chemokine ligand 10), and ITGAX (integrin subunit alpha X).

**Table 5:** Top down and up-regulated hub genes identified with high degree

Gene_Symbol	Ensemble gene ID	Degree	Description
Up-regulated hub genes			
NEUROD1	ENSG00000162992	8	neuronal differentiation 1
ATP4A	ENSG00000105675	6	ATPase H+/K+ transporting subunit alpha
DCX	ENSG00000077279	6	doublecortin
SLC10A2	ENSG00000125255	4	solute carrier family 10
ACKR1	ENSG00000213088	4	atypical chemokine receptor 1
KEL	ENSG00000197993	4	Kell metallo-endopeptidase
HCK	ENSG00000101336	4	HCK proto-oncogene
OMD	ENSG00000127083	4	osteomodulin
SCEL	ENSG00000136155	4	sciellin
CALB2	ENSG00000172137	4	calbindin 2
Down-regulated hub genes			
IL1B	ENSG00000125538	36	interleukin 1 beta
ITGAM	ENSG00000169896	31	integrin subunit alpha M
CXCL8	ENSG00000169429	30	C-X-C motif chemokine ligand 8
CXCL10	ENSG00000169245	28	C-X-C motif chemokine ligand 10
CCL5	ENSG00000271503	27	C-C motif chemokine ligand 5
ITGAX	ENSG00000140678	25	integrin subunit alpha X
CXCL11	ENSG00000169248	24	C-X-C motif chemokine ligand 11
CSF3	ENSG00000108342	21	colony stimulating factor 3
CCR7	ENSG00000126353	21	C-C motif chemokine receptor 7
SELL	ENSG00000188404	21	selectin L

### Long non‑coding RNAs (LncRNAs)

Top up and down-regulated lncRNAs in temozolomide-resistant glioblastoma cell lines are shown in Table 6. The up-regulated lncRNAs including NAV2-AS2, CAMTA1-AS2, MEIS1-AS3, and CSE1L-DT and down-regulated ncRNAs such as MIR3142HG, SUGCT-AS1, DBH-AS1, LIX1-AS1, and SWINGN were identified.

**Table 6:** Top up and down-regulated LncRNAs in temozolomide-resistant glioblastoma cell lines

Up-regulated lncRNA	log2(FC)	Ensemble gene ID	Down-regulated lncRNA	log2(FC)	Ensemble gene ID
NAV2-AS2	5.36	ENSG00000254453	MIR3142HG	-5.02	ENSG00000253522
CAMTA1-AS2	5.22	ENSG00000237728	SUGCT-AS1	-4.57	ENSG00000203446
MEIS1-AS3	1.9	ENSG00000226819	DBH-AS1	-3.9	ENSG00000225756
CSE1L-DT	3.05	ENSG00000227431	LIX1-AS1	-4.30	ENSG00000260910
OXR1-AS1	2	ENSG00000253582	SWINGN	-4.17	ENSG00000260910
DOCK4-AS1	3.2	ENSG00000225572	SIRLNT	-3.8	ENSG00000253802
KCNQ5-AS1	2.11	ENSG00000229154	CASP4LP	-3.04	ENSG00000291173
LNX1-AS1	3.5	ENSG00000250930	CEDORA	-2.7	ENSG00000260788
TRPS1-AS1	3.3	ENSG00000227170	CRMA	-2.7	ENSG00000174403
LINC02327	3.46	ENSG00000258038	DDX3ILA1	-2.6	ENSG00000232756

### Validation of key genes through reported transcriptomics studies

**Fig. 5:**
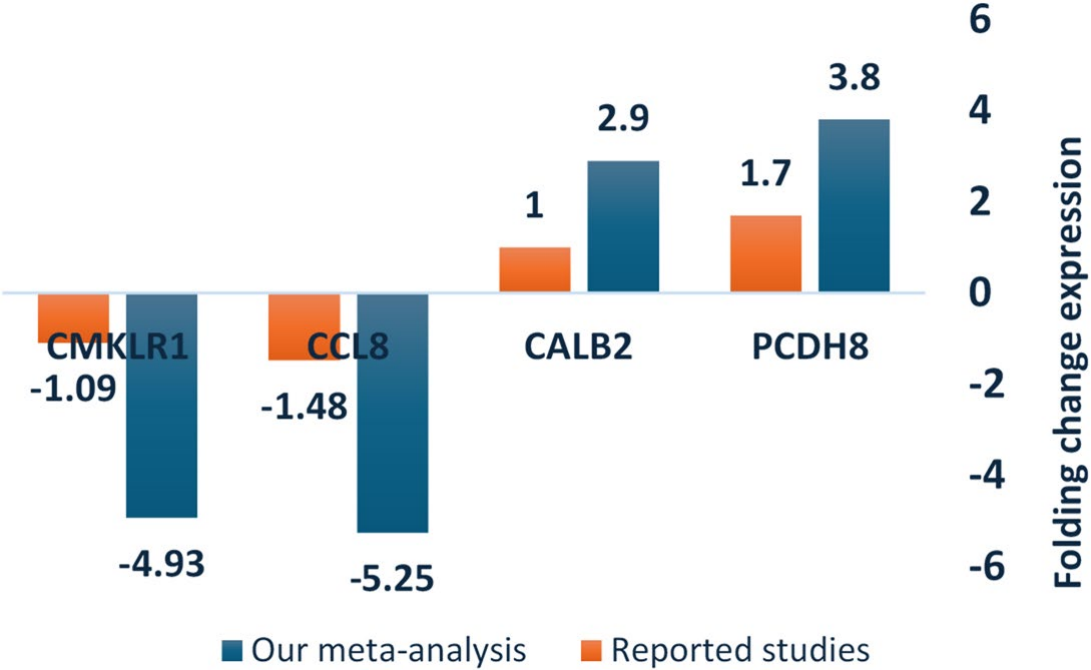
Comparison of up and downregulated genes with reported qRT-PCR studies

According to the expression level of the identified key genes related to TMZ glioblastoma cell lines including PCDH8 (protocadherin 8), CALB2 (calbindin 2), CCL8 (C-C motif chemokine ligand 8), and CMKLR1 (chemerin chemokine-like receptor 1) were compared with reported qRT-PCR studies (Fig. 5). Based on this, it can help to some extent confirm the results of this research.

## Discussion

TMZ is the effective chemotherapeutic agent and act by damaging DNA and suppressing proliferation in glioblastoma [25]. The existence of blood-brain barrier hinders the effective accumulation of TMZ in the brain, further compromising its efficacy in treating glioblastoma [26]. Glioblastoma is the most common and deadly form of primary brain cancer. The metalloprotease meprin β (Mep1b) is capable of cleaving cell-adhesion molecules and plays a role in regulating brain endothelial tight junction (TJ) proteins and therefore affecting blood–brain barrier (BBB) tightness in vitro and in vivo [27]. Tandem C2 domains nuclear protein (TC2N) is a tandem C2 domain-containing protein that is differentially expressed in several types of cancers and is closely associated with tumorigenesis and tumor progression. TC2N has been identified as an oncogene in lung and gastric cancer and may as a potential target for the diagnosis and treatment of different types of cancers [28]. Sialic acid binding Ig like lectin 15 (SIGLEC-15) has emerged as a pivotal immunosuppressive mediator and a promising therapeutic target in cancer immunotherapy [29]. C-C motif chemokine ligand 8 (CCL8) is a Tumor-associated macrophages (TAMs)-associated factor to mediate invasion and stemness of glioblastoma, and targeting CCL8 may provide an insight strategy for glioblastoma treatment [13]. Myelin oligodendrocyte glycoprotein (MOG) is located on the myelin sheath outer surface and has an extracellular domain, which makes it accessible to potential antibodies. The human MOG gene is located in the major histocompatibility complex (MHC) locus on chromosome 6p21.3-p22 and is a member of the immunoglobulin superfamily [30]. In our study, some key genes with high expression level involved in glioblastoma included NeuroD4, IgSF21, PCDH8, ACKR1, Mep1b, and TC2N as up-regulated genes and ITGAX, SIGLEC-15, CCL8, MOG, PENK, SELE, and PIGR as down-regulated genes (Table 2).

GO is a major bioinformatics initiative that provides a standard vocabulary for describing gene and gene product characteristics across all species. Atonal bHLH transcription factor 1 (ATOH1) plays an important role in neural development and may serve as a tumor suppressor or an oncogene as well as it regulates neurogenesis in the cerebellum [31]. Glucose-regulated protein 78 (GRP78) regulates multiple signaling pathways and could be used as a biomarker for high-grade glioma [32]. The hematopoietic cell kinase (HCK) stimulates cell proliferation and plays a role in chemo resistance and reduced drug efficacy in clinical study and may be served as a promising therapeutic target for glioblastoma [33]. In our study, the up-expressed genes including NEUROD and ATOH1 in BP; GPR78 in MF; and HCK in CC as key genes were identified (Fig. 3). Next, interleukin 11 (IL 11) gene is a pleiotropic cytokine has been shown to increase tumour proliferation and can be considered a prognostic biomarker in different types of cancer which is secreted by glioblastoma cells [34]. CD74 has been shown to function as a chaperone in the transport of MHC II molecules, which are involved in antigen presentation and its activation may have potential in tumor immunotherapy [35]. Furthermore, in this study down-expressed genes including IL11, and CCL24, and ZBP1 in BP; CXCL10, and CCL8 in MF; and CD74, and HLA-DMB genes in CC as key genes were identified (Fig. 3).

According to KEGG database, key genes including CCL5, IL6, and IL7 genes related to cytokine-cytokine receptor interaction pathway; IL2, CXCL10, IL4, CNTF, CTF1, CLCF, and CSF1/2 genes associated with JAK-STAT signaling pathway were identified (Table 3). Cytokines including interleukins, interferons, tumor necrosis factors, C-C motif chemokine ligand 2 (CCL2), and CCL5 are critical in regulating immune responses and can promote or inhibit tumor growth, influence the tumor microenvironment, and impact the efficacy of cancer treatments [36]. The JAK/STAT signaling pathway plays a pivotal role in mediating the effects of CXCL10 signaling, which promotes the expression of pro-inflammatory cytokines and chemokines, thereby driving the proliferation, differentiation and activation of various immune cells [37]. Moreover, IL6, CXCL20, CXCL8 and IL1B genes belonged to IL-17 signaling pathway; CASP1, HRK, and TRAF1 genes belonged to apoptosis; and VAV3, COL1A1 and CDKN1A with cell adhesion function related to proteoglycans in cancer were identified (Table 3). IL-17, a potent proinflammatory cytokine, has been shown to intimately contribute to the formation, growth, and metastasis of a wide range of malignancies and recent studies implicate IL-17 as a link among inflammation, and cancer. Cell adhesion molecules regulate glioblastoma cell invasion and drug resistance [38].

Most cancer-causing signaling pathways converge on a set of TFs that ultimately control gene expression patterns, leading to tumor formation, metastasis, and progression [39]. Aberrant expression of TFAP-2 Gamma (TFAP2C) serves as a prognostic factor for cancer patients and therapeutic target to attenuate chemoresistance of cancers [40]. Transcription factor B-cell lymphoma/leukemia 11 A (BCL11A) gene is predominantly expressed in brain and its function mainly as a transcriptional repressor that is crucial in brain [41]. IRF3 acts as an important regulator of LPS-mediated neuroinflammatory responses and highlight IRF3 as a central regulator of disease-specific gene activation in different neuroinflammatory diseases [42]. Moreover, leu-rich_rpt, and BHLH as up-regulated domains and EGF_2, and chemokine_IL8 as down-regulated domains were identified. Leucine-rich repeat (LRR) plays a significant part in the formation and differentiation of synapses, and neurotransmitter transmission and are regulated in cancers which is a potential tumor suppressor gene [43]. In our study, TFAP2C, and BCL11A as up-regulated TFs and IRF3, and CEBPB as down-regulated TFs were identified (Table 4).

Hub genes are defined as genes that have a large number of connections or interactions with other genes in a regulatory network. In our study, up-expressed genes including NeuroD1, DCX, and ACKR1 and down-regulated genes such as IL-1β, CXCL10, and ITGAX with high degree as key hub genes were identified (Table 5; Fig. 4A). NeuroD4 (Neuronal Differentiation 4) in neuronal development has the potential to convert astrocytes into functional neurons, thus offering a potential novel therapeutic approach for glioblastoma. NeuroD1 expression is retained in the cerebral cortex, and it overrides oncogenic mutations present in medulloblastoma cells [44]. Doublecortin (DCX) is a brain-specific gene and has recently emerged as a potential player in cancer progression as well as has been reported that DCX synthesis induces apoptosis in BTSC through a novel JNK1/neurabin II/DCX/PP1/caspase‐3 pathway [45]. Interleukin-1 beta (IL-1β) is the major driving force in neuroinflammation and is produced by immune cells, fibroblasts, and metastasis in glioblastoma cancer pathogenesis [46]. C-X-C motif chemokine ligand 10 (CXCL10) is a chemokine crucial for immune cell recruitment and inflammation modulation serve as targets for novel therapeutic strategies in neurological disorders which is expressed by neurons, and astrocytes [37]. The integrin subunit genes (ITGs) roles have been reported in tumorigenesis as potential prognostic biomarkers and ITGAX may play a critical role in cancer progression by modulating the epithelial-mesenchymal transition (EMT) pathway which its overexpression enhances cell proliferation, and tumorigenic [47]. Overall, these hub genes play a crucial role in understanding the complexity and functionality of genetic networks.

LncRNAs have a critical function in various physiological processes and pathological conditions including tumor formation, malignant transformation, apoptotic cell death, epigenetic modification, genomic imprinting, spermatogenesis regulation, and maturation [48]. Glioblastoma is an aggressive malignant cerebral tumor and the exact molecular mechanism of this type of cancer is not clear. The increasing levels of several tumorigenic lncRNAs have been identified in glioblastoma. In our study, key up-regulated ncRNAs including NAV2-AS2 (log2FC = 5.36), CAMTA1-AS2 (log2FC = 5.22), MEIS1-AS3 (log2FC = 1.9), and CSE1L-DT (log2FC = 3.85) were identified (Table 6). Calmodulin-binding transcriptional activator 1 (CAMTA1) is highly expressed in the brain and plays a role in cell cycle regulation, cell differentiation, regulation of long-term memory, and initial development, maturation, and survival of cerebellar neurons [49]. NAV2-AS2 is a lncRNA that is located on chromosome 2 and is believed to play a role in regulating gene expression and have shown that it is upregulated and its potential involvement in tumorigenesis [48]. Moreover, key down-regulated lncRNAs such as MIR3142HG (log2FC= -5.02), SWINGN (log2FC= -4.17), LIX1-AS1 (log2FC= -4.30), SUGCT-AS1 (log2FC= -4.57), and DBH-AS1 (log2FC= -3.9) were identified (Table 6). SWINGN is implicated in cancer by acting as a regulator of gene expression and potentially contributing to tumor progression [50]. Studies have demonstrated that genetic variants in the miRNA-coding genes might be associated with cancer susceptibility and survival. The impact of rs1582417, rs17057846, rs2431689, rs2961920, rs58747524, and rs7727115 polymorphisms in MIR3142HG on the susceptibility to prognosis of glioma in the Chinese Han population has been provided [51]. Macrophages are the major primary immune cells that mediate the inflammatory response. SUGCT-AS1 is a human macrophage-specific lncRNA whose expression is increased upon M1 macrophage stimulation. Conditioned media of SUGCT-AS1-depleted M1 macrophages induced an inflammatory phenotype of vascular smooth muscle cells, which included increased expression of inflammatory genes (IL1B and IL6), as well as is a novel inflammatory regulatory mechanism in macrophages [52]. DBH-AS1 can be induced by hepatitis B virus x protein (HBx) and inactivated by p53, and consequently promote cell proliferation and cell survival through activation of MAPK signaling in hepatocellular carcinoma (HCC) and finally acts as an oncogene for HCC. DBH-AS1 facilitated the development of HCC via miR-138/FAK/Src/ERK pathway, establishing the molecular basis of DBH-AS1 in clinical application for HCC [53].

For validation checking, a comparison was made on the gene expression level based on the fold changes of key genes including PCDH8 [54], CALB2 [55], CCL8 [56], and CMKLR1 [57] with results of previous qRT-PCR (Fig. 5). Future in vitro and in vivo studies are required to validate these key genes related to TMZ Glioblastoma cell lines through molecular and biotechnology techniques.

## Conclusion

In conclusion, RNAseq technology has allowed us to simultaneously examine the expression of thousands of genes and investigation of molecular mechanism related to temozolomide-resistant glioblastoma cells. Glioblastoma cells showed different gene expression compared to normal tissues. In an overview, the key genes obtained from this research, after clinical and molecular tests as experimental validation with positive results, they can be suggested as influential genes associated with temozolomide-resistant glioblastoma cell lines.

## Limitation

Although our analysis provides good and significant results for researchers in the field of bioinformatics and gene expression for the treatment of glioblastoma cells, we must acknowledge one important limitation. limitation is related to the lack of real-time PCR to confirm the highly expressed hub genes.

## Electronic Supplementary Material

Below is the link to the electronic supplementary material.

Supplementary file 1.

## Data Availability

The data of Table 1 that support this study are available in NCBI GEO site at [https://www.ncbi.nlm.nih.gov/geo/] (https:/www.ncbi.nlm.nih.gov/geo) .
