# Supplementary material for: Identification of hub mRNAs and long non-coding RNAs involved in temozolomide-resistant glioblastoma (brain cancer) cell lines
Source: Discov Oncol. 2026 Jun 5;17:1145. doi: 10.1007/s12672-026-05243-2 (PMC13457988; doi:10.1007/s12672-026-05243-2)
Supplement: Supplementary file 1 [file 12672_2026_5243_MOESM1_ESM.docx]

Supplementary file

Supplementary file fig1. Cluster dendrogram plot after normalization of RNAseq data
